# From Structure to Optics: The pH-Temperature Interplay in Aqueous Solution CdS Nanoparticles

**DOI:** 10.3390/nano16010003

**Published:** 2025-12-19

**Authors:** Elvia Angelica Sanchez-Ramirez, Ramón Arellano-Piña, M. A. Hernandez-Perez, Simón Bello-Teodoro, Karol Karla Garcia-Aguirre, J. Sastré-Hernández, J. R. Aguilar-Hernandez

**Affiliations:** 1 Departamento de Ingeniería Metalúrgica-UPIIZ, Instituto Politécnico Nacional, Zacatecas CP 98160, Mexico; larellanop@ipn.mx; 2 Departamento de Ingeniería en Metalurgia y Materiales, ESIQIE, Instituto Politécnico Nacional, Ciudad de Mexico CP 07738, Mexico; mahernandezpe@ipn.mx; 3 Unidad Académica de Ingeniería I, Departamento de Metalurgia Extractiva, Universidad Autónoma de Zacatecas “Francisco García Salinas”, Zacatecas CP 98160, Mexico; simon.bello276@uaz.edu.mx; 4 Departamento de Bioingenieria, Unidad Professional Interdisciplinaria de Ingeniería Campus Zacatecas, Instituto Politécnico Nacional, Zacatecas CP 98160, Mexico; kgarciaa@ipn.mx; 5 Departamento de Física, Escuela Superior de Física y Matemáticas, Instituto Politécnico Nacional, Edificio 9, UPALM, Col. Lindavista, Ciudad de Mexico CP 07738, Mexico; jsastre@ipn.mx (J.S.-H.); jaguilarh@ipn.mx (J.R.A.-H.)

**Keywords:** CdS nanoparticles, pH effect, semiconductor materials, optical properties, structural properties, aqueous medium

## Abstract

Cadmium sulfide (CdS) nanoparticles are classified as II-VI semiconductor materials, used in optoelectronic devices due to a band gap (E_g_). In this study, CdS nanoparticles were synthesized by the chemical precipitation method, and a systematic evaluation of pH (4.8–10.1) and temperature (50, 75, and 90 °C) was conducted. The effects of these variables were evaluated by UV-VIS spectroscopy, X-ray diffraction (XRD), and scanning electron microscopy (SEM). Results demonstrate that variables determine crystallite sizes (C_s_), cluster sizes, and optical properties. CdS crystallization is more affected by pH conditions than by temperature during synthesis; the change in peak intensity in 2θ = 24–29° suggests the formation of a cubic phase (alkaline conditions) and a transition to a hexagonal phase (acidic conditions). Higher temperature improves the quality of the nanoparticles, as evidenced by the reduction in intensity of the peaks associated with secondary materials. The synthesis conditions of CdS nanoparticles significantly affect E_g_, widening the range from 2.21 to 2.40 eV. Both temperature and pH conditions change the size of nanoparticles and clusters. Acid conditions promote the formation of rounded and uniform nanoparticles, while alkaline conditions form the largest crystals of CdS. These findings are useful for developing electronic devices that require different semiconductor profiles.

## 1. Introduction

Semiconductor nanoparticles are in the scale of 1 to 100 nm [1] that can be prepared by different methods, such as sol–gel [2], hydrothermal [3], chemical precipitation [4,5,6], solvothermal [7], green biosynthesis [8,9], wet chemical [1,10], ultrasonic wave irradiation [11] and coprecipitation [12]. In recent years, the study of II-VI semiconductors has experienced significant growth as these materials exhibit tunable properties and different morphologies, such as quantum dots, nanorods, nanotubes, nanospheres, and nanowires, among others [13,14], making them highly promising for various material applications. Specifically, cadmium sulfide (CdS) nanoparticles have attracted considerable attention in recent decades due to their optical, mechanical, magnetic, and electrical properties [11], which lie between those of bulk and quantum-sized materials [1]. These properties make CdS suitable for applications such as solar cells, photodetectors [10], light-emitting diodes, photocatalytic processes, and, more recently, hydrogen generation and in phytoremediation for the removal of heavy metals from soil and water [15,16,17]. Moreover, CdS has the potential to form hybrid materials by the combination of biotechnology and nanotechnology [3].

One of the most remarkable features of these nanomaterials is that their properties can be tuned by varying synthesis conditions, allowing control over particle size, morphology, crystalline phase, and band gap energy. Among the synthesis parameters that influence the final properties of CdS, the pH of the solution plays a key role. Variations in this parameter affect the kinetic rate, nucleation rate, colloidal stability, crystal growth, and surface area-to-volume ratio, leading to significant changes in particle size, shape distribution, and the intensity of optical transitions [11].

Different chemical species have been explored as a precursor of the anions or cations, such as cadmium sulfate [4,18], cadmium acetate [8], cadmium nitrate [19], and cadmium chloride [6,7], to form the structural material, as well as several compounds that have been employed as buffer solution and ligands as well as potassium hydroxide (KOH) [6], sodium hydroxide (NaOH) [6,10], ammonium hydroxide (NH_4_OH), ethylenediamine C_2_H_8_N_2_ [20], citrate complex [21,22], triethanolamine ${(C}_{6}H_{15}{\mathrm{NO}}_{3})$, and tartaric acid C_4_H_6_O_6_. It is known that the formation of complexes is affected by the concentration of hydronium or hydroxyl ions, since there is the possibility of forming different types of cadmium complexes; it has been reported that there are six types of amino–cadmium complexes that are $Cd(NH_{3}{)}^{2+},\;Cd(NH_{3}{)}_{2}^{2+},\;Cd(NH_{3}{)}_{3}^{2+},\;Cd(NH_{3}{)}_{4}^{2+},\;Cd(NH_{3}{)}_{5}^{2+}$ and $Cd(NH_{3}{)}_{6}^{2+}$ [23].

The concentration and type of reactants are crucial parameters, as they influence precipitation kinetics and thus characteristics such as size and morphology [24]. Several studies support the above; for example, some researchers reported that acidic conditions favor nanoparticles growth by an ion–ion mechanism [21], which has a cubic phase and a nanometric dimension [19], while alkaline medium promotes the hydroxide aggregation mechanism [21], and nanoparticles growth in a hexagonal phase [6,11,12]. The synthesis technique determines the nanoparticles’ characteristics; relevant aspects in an aqueous medium include scalability, reproducibility, the simplicity of the equipment, and the low process temperature. Despite these advances, it is still necessary to further explore the relationships among pH, temperature, and the structural and optical properties of CdS nanoparticles, as such variations can influence their efficiency in specific technological applications.

In this work, we present the synthesis of CdS nanoparticles by the chemical precipitation method and a systematic study of their synthesis under different pH and temperature conditions. Covering a wide range from acid medium to basic medium, as well as their characterization through structural and spectroscopic techniques, to establish how this dual parameter pH and temperature approach offers a scalable and straightforward route to tailor the semiconductor between effects on the final characteristics of CdS nanoparticles. These findings offer an accessible framework to tailor the structural–optical relationship in CdS nanoparticles through parameter tuning, providing fundamental insights relevant for the design of nanoengineered semiconductor systems and their optimization for technological applications.

## 2. Materials and Methods

### 2.1. CdS Nanoparticles Synthesis

Cadmium sulfide (CdS) nanoparticles were synthesized by a chemical precipitation method, maintaining constant the reaction time in 30 min and under controlled pH and temperature conditions; all solutions were prepared using Fermont analytical grade chemical species of cadmium chloride (CdCl_2_) and thiourea (CH_4_N_2_S) 0.1 M aqueous solutions as precursors of cadmium (Cd) and sulfur (S), respectively, were prepared and mixed under continuous stirring at 240 rpm until reaching the reaction temperature. A similar procedure was explored to grow thin films of CdS [25]. The pH range studied from 4.8 to 10.1 was monitored using a potentiometer, Horiba PC110 (Kyoto, Japan). To explore these values of pH, the reaction medium was adjusted using NH_4_OH and NH_4_Cl to generate a medium and a buffer solution, and a Cd-complex to control the reaction rate. In the case of the acid reaction, hydrochloric acid (HCl 0.1 M) was used, because it is an efficient reactant to control the pH through the H^+^ released in solution, and is economical and versatile. The synthesis was conducted at temperatures ranging from 50 °C to 90 °C to evaluate their combined effect on nanoparticle formation. Reaction temperature was sensed with a PID-controlled thermocouple attached to an IKA heating plate (Staufen, Germany). After completion of the reaction, the resulting precipitates were filtered and washed thoroughly with distilled water to remove residual ions or unreacted species, and then dried at room temperature for 24 h to avoid undesired morphological or structural changes potentially caused by thermal drying. This approach helps preserve the synthesized characteristics of the CdS nanoparticles, preventing aggregation and phase instability during aqua evaporation [26]. No characterization data are reported for CdS nanoparticles synthesized at 4.7 and 50 °C by XRD and UV-Vis, as only a small amount was obtained, insufficient for analysis.

### 2.2. Characterization

The obtained samples were characterized using various analytical techniques to determine their structural, morphological, and optical properties, including X-ray diffraction (XRD), employing a Bruker Advanced diffractometer (Cu *K*α = 1.5406 Å) in a *θ*/*2θ* configuration (2*θ* = 20–60°) to evaluate crystallinity and phase structure. Crystallite size was calculated according to Scherrer’s equation, $C_{s}=\frac{k\lambda }{Bcos\;\theta }$, where *C_s_* crystallite size and k Scherrer constant for spherical crystals is about 0.9, λ = X-ray wavelength 1.5406 Å, *B* = full width at half maximum (FWHM) of the peak, and θ = Bragg angle.

Optical absorption was measured using a Perkin-Elmer Lambda 35 UV-Vis spectrometer. CdS nanoparticles were placed between two Corning glass slides and subjected to UV-Vis analysis, diffuse reflectance, and transmittance methods rather than being suspended in solution. To estimate the E_g_, the first derivative of the optical density was used through (d[OD]/dE), with respect to photon energy (eV). This approach finds E_g_ at the energy level corresponding to the absolute maximum or minimum in the derivative curve, which specifies the onset of interband electronic transitions.

This method is especially convenient when the samples show light dispersion, overlapping absorption peaks from defects, or when the linear region required for a conventional Tauc plot analysis is difficult to distinguish visually. Multiple studies have successfully used derivative analysis in CdS and other nanometric semiconductors, demonstrating good agreement with classical methods. Consequently, given the colloidal nature and the presence of surface characteristics/defects in our samples, we consider that the derivative of the optical density provides a robust and reproducible estimate of E_g_ for comparative purposes in this study [27,28].

Scanning electron microscopy (SEM) was used to study the surface morphology of the nanoparticles with an SEM JEOL JSM6701F. The probability density of the approximate diameters of the CdS clusters for all treatments. The data were obtained by measuring at least 250 CdS clusters per treatment to achieve a 90% confidence level. These measurements were performed using scanning electron microscopy (SEM) imaging at a magnification of 60,000×.

## 3. Results and Discussion

The results of the synthesis and characterization of CdS nanoparticles prepared by the chemical precipitation method under varying pH and temperature conditions are presented in this section. The influence of these parameters on the structural, morphological, and optical properties of the samples was systematically evaluated. The experimental data revealed that both pH and temperature significantly affected the growth mechanism, particle size, crystallinity, and optical behavior. To explain these effects, the following sections describe the main findings obtained from X-ray diffraction, UV-Vis spectroscopy, and SEM analyses.

**Figure 1:**
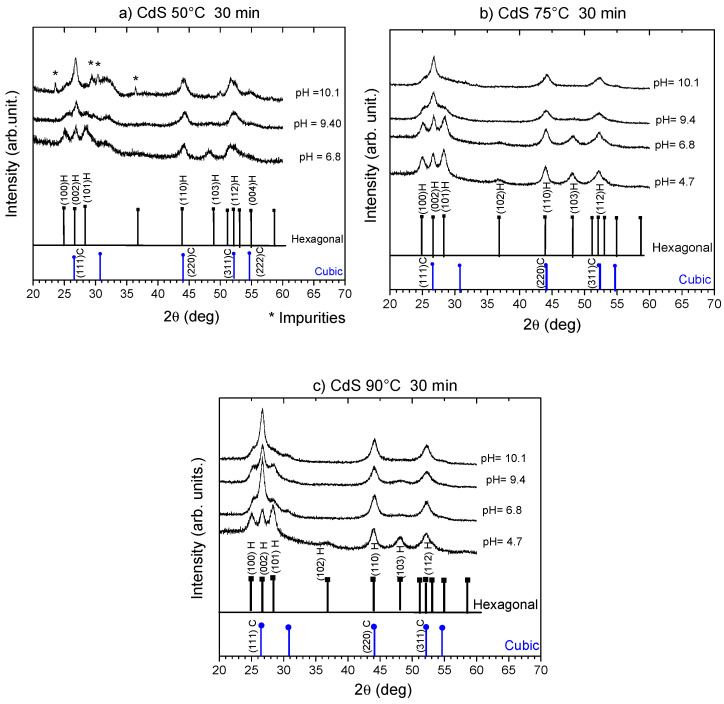
XRD spectra of CdS nanoparticles obtained at (**a**) 50 °C, (**b**) 75 °C, and (**c**) 90 °C for different pH values. XRD patterns of CdS cubic and hexagonal phases are included in each graph. * impurities of CdCO_3_.

Figure 1 shows the XRD patterns of CdS nanoparticles obtained at 50 °C, 75 °C, and 90 °C during 30 min, at different pH levels (4.7–10.1). In general, as expected, nanoparticles are polycrystalline. It has been reported that this material can crystallize in the zinc blende [4,29], wurtzite [1,7,9], or a mixture of both phases [6] and that a transition between these phases occurs [5]. The spectra show several peaks that correspond to (100), (002), (101), (102), (110), (103), and (112) planes of hexagonal phase according to JCPDS No. 800006, and some peaks could also be related with cubic phase and the (111), (220) and (311) planes in accordance with JCPDS 800019. In Table 1, the peak positions that correspond to these planes are presented. pH is a parameter that could be influenced by the predominant crystalline phase in materials obtained by chemical methods. In our case, a possible explanation is the kinetics of thiourea decomposition, since hydrolysis is sensitive to pH, temperature, and the catalytic activity at the surface of the solids [30]. For example, if the decomposition occurs in an acidic medium, the oxidation product $\left[{\left({NH}_{2}\right)}_{2}\right]CO$ (Equation (1)) can form, which promotes the release of S, particularly at room temperature, due to the solubility of HS; this is important because temperature influences solubility [31]. On the contrary, under basic conditions, the reaction leads to the release of S^−2^, but different products $H_{2}S,\;SH^{-},\;S^{2-}$ (Equations (2) and (3)) are formed depending on the reaction temperature. Another factor could be thermal decomposition, which increases the possible route chemical (Equations (4)–(6)).

Acidic medium [32]:

(1) $$\left[{\left({NH}_{2}\right)}_{2}\right]CS\;+2H_{2}O+\;H↔\left[{\left({NH}_{2}\right)}_{2}\right]CO\;+\;H_{2}S$$

Alkaline medium [30]:

(2) $$\left[{\left({NH}_{2}\right)}_{2}\right]CS\;+OH^{-}↔\;SH^{-}+\;H_{2}O\;+\;{CN}_{2}H_{2}$$

(3) $$SH^{-}+{OH}^{-}↔\;S^{2-}+H_{2}O$$

Thiourea thermal decomposition [32]:

(4) SC(NH_2_)_2(__aq)_ ↔ H_2_S _(aq)_ + H_2_NCN _(aq)_

(5) H_2_NCN_(aq)_ ↔ HNCN^−^(aq) + H^+^_(aq)_

(6) NCNH^−^_(aq)_ ↔ NCN^2−^_(aq)_+ H^+^_(aq)_

Several reactions occur at the same time in the bath as the quantity of the reactants increases, and the complexity and kinetics of the reactions change. Both pH and temperature alter the growth kinetics, mainly because (1) the hydrogen ions (H^+^) concentration directly influences the precipitation processes. A higher amount of H^+^ hinders the precipitation of CdS, reducing the release and, therefore, the concentration of S, and (2) cadmium species in aqueous solution from the free ion, cadmium hydroxide, and until cadmium complexes form Cd(NH_3_)_x_^2+^.

For nanoparticles CdS synthetized at pH = 10.1 at 50 °C, some non-CdS peaks are located at 2*θ* = 23.47°, 30.3°, 36.43°, and 50.04°, which are related to CdCO_3_ of the hexagonal phase, reported in the reference (PDF 42–1342, ICSD 20181) [33], due to the reaction complexity and interactions between these kinds of species in alkaline medium.

CdS nanoparticles obtained in this research at pH 6.8 exhibit 7 planes characteristics of the hexagonal phase. As pH increases, a decrease in intensity on the (100) and (101) planes is observed, favoring the preferential orientation on the (002) plane of the hexagonal phase or the (111) plane of the cubic phase. Similar behavior is seen for nanoparticles obtained at 75 and 90 °C, except that in these conditions, no impurities are detected. On the other hand, the reduction in the intensity of some peaks until they disappear suggests two possibilities: (1) total transformation from hexagonal to cubic phases influenced by pH, or (2) a change in the proportion of both phases, suggesting that these materials are composed of a mixture of the phases. Moreover, the peak intensity is not the same as reported in XRD files; from this data, it is not possible to determine the proportions corresponding to each phase. This change is observed in all temperatures for pH values above 9.5, and these results agree with [30]. Consistent with these results, pH strongly influences crystallinity because the kinetics of formation are controlled by rapid hydrolysis, the release rate of Cd^2+^, and the dominance of the hydroxide mechanism, which favors the cubic phase.

The C_s_ was estimated using the Scherrer equation from the analysis of *B* (FWHM) of the peak related to the (002) plane (i.e., (111)). In addition, three peaks were observed between 24 and 29°; in these cases, the average value for each peak was calculated. C_s_ oscillates around 7–11 nm for all samples, similar to the values obtained by chemical methods [34,35,36], such as the hydrothermal process [3], as shown in Table 1. Therefore, pH does not affect C_s_.

**Figure 2:**
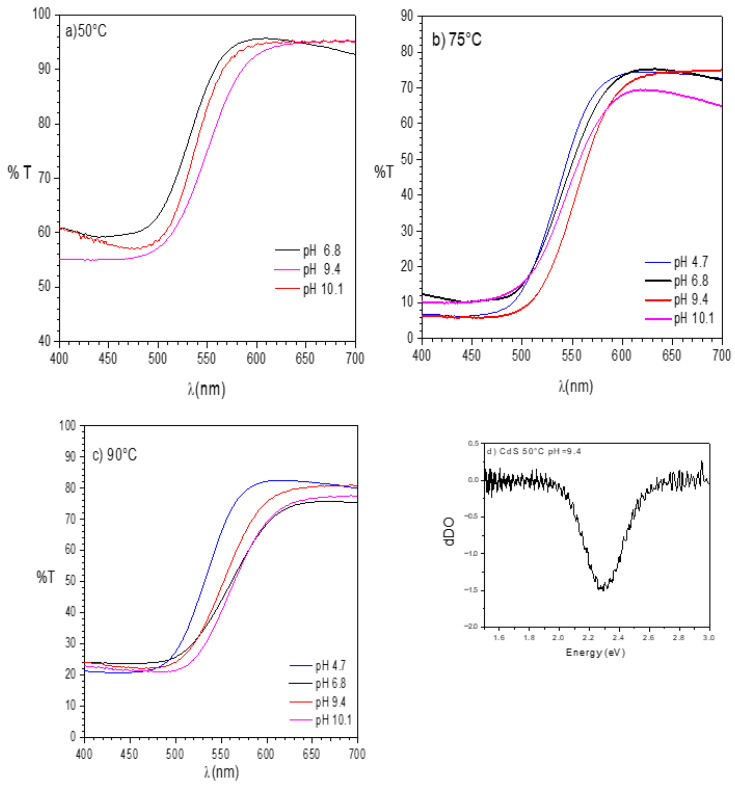
Transmittance spectra of CdS nanoparticles synthesized at different pHs, (**a**) 50 °C, (**b**) 75 °C, and (**c**) 90 °C, (**d**) first derivative.

As can be seen in Figure 2a–c, a well-defined absorption edge for all the samples is obtained. From the samples at 75 °C, it is clear that there is a strong dependence of the pH value on the displacement of the absorption edge.

Also, high transmittance values were observed for all sets of samples, exceeding 70%. These results are comparable to those reported by [12,29,37], which are suitable for optoelectronic and optical applications. Based on these experimental results, the band energy values, E_g_, were calculated from the first derivative of the optical density (Figure 2d) and summarized in Table 1. Some values are consistent with those reported in bulk materials and in CdS thin films [18,38].

The pH significantly affects the E_g_ values, showing an inverse relationship [11,39,40]. The sensitivity of E_g_ to pH variations increases progressively with temperature, as can be seen at 90 °C, where it ranges from 2.39 to 2.21 eV. The shift in the E_g_ value can be related to the temperature-induced lattice expansion and the enhancement of atomic vibrations, as higher temperatures increase the kinetic energy of the atoms, leading to greater vibrational amplitude. Another possible explanation is a variation in cell volume, due to increased strain in the lattice of CdS nanoparticles. Therefore, the Eg value would not be influenced by crystallite size, as it had remained almost constant at 7–10 nm for all synthesis conditions. These values were approximately twice the Bohr radius exciton of CdS, which has been reported to be between 5 and 6 nm.

**Figure 3:**
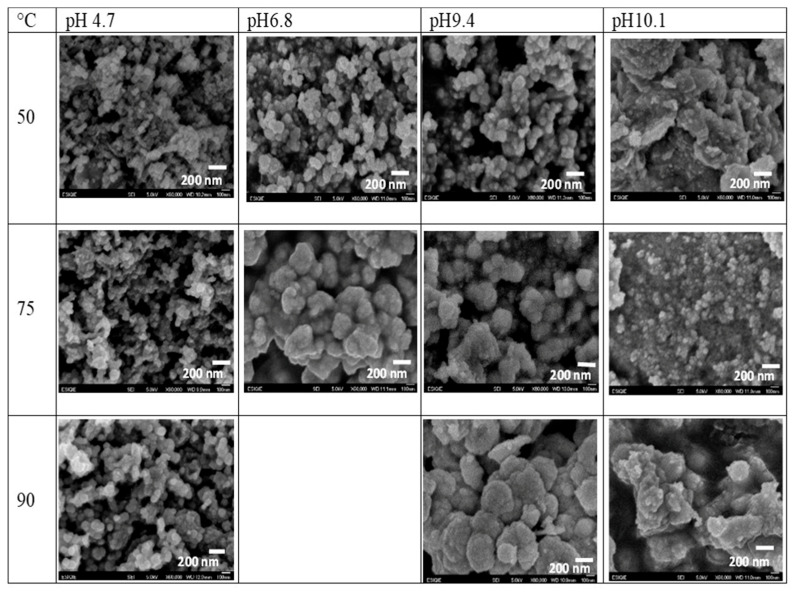
Micrographs of CdS nanoparticles synthesized at different conditions of pH and temperature.

Figure 3 shows micrographs of the CdS nanoparticles synthesized under different pH and temperature conditions, showing changes in cluster size. In general, CdS nanoparticles exhibit a hemispherical agglomerate morphology under all conditions studied. The CdS cluster size is more sensitive to pH changes than to temperature changes during synthesis. At a pH between 4.7 and 6.8, clusters of nanoparticles with sizes between 20 and 80 nm will form (a probability of at least 70% with 90% confidence). In contrast, at alkaline pH, the growth of larger CdS clusters is promoted, resulting in only 12% of the particles staying in the 20–80 nm size range.

On the other hand, the temperature during synthesis does not have a strong influence on the cluster size; despite the wide range of temperatures evaluated, there is only a slight change in the cluster diameters (further details on cluster size are reported in the Supplementary Materials). The bigger sizes were acquired close to the boiling temperature of precursor aqueous solutions; the increase in size can be related to the quantity of energy promoting the growth of clusters.

At low pH, the concentration of S^2−^ ions decreases due to protonation (HS^−^ formation), which slows nucleation. Particles smaller than the critical radius are unstable and redissolve via intermediate chemical species rather than continuing to grow. Particle size decreases with increasing pH, suggesting faster nucleation under basic conditions. At 50 °C, the nucleation rate occurs faster than growth, and is likely a known phenomenon, such as burst nucleation. The competition between nucleation and growth rates determines the final particle size. A more uniform morphology indicates higher supersaturation and controlled nucleation; this mechanism is observed at higher temperatures and pH.

## 4. Conclusions

The results demonstrated that both parameters play a critical role in determining the material’s structural, morphological, and optical properties. The pH of the synthesis medium critically governs the phase evolution of CdS nanostructures. Acidic conditions suggest stabilization of the hexagonal wurtzite phase due to limited dissociation of sulfide species, leading to slower nucleation kinetics. In contrast, an alkaline medium enhances the supersaturation of S^2−^, promoting a structural transition toward the cubic form or the growth of a mixture of both phases.

SEM observations confirm that acidic media and lower temperatures result in smaller, irregular nanoparticles. In contrast, higher pH values and elevated temperatures favored the formation of larger and more uniform grains. These effects are attributed to the interplay between thermally activated diffusion processes that drive recrystallization and particle coalescence. The optical characterization also exhibits a clear correlation between the synthesis conditions and the band gap energy. As particle size increased with temperature and alkalinity, the band gap decreased gradually due to reduced quantum-confinement effects. Therefore, by controlling pH and temperature, it is possible to fine-tune the crystallographic structure and optical response of CdS nanoparticles, offering a simple yet versatile strategy to improve their performance in optoelectronic and photocatalytic applications.

## Figures and Tables

**Table 1:** C_s_ and E_g_ values of CdS nanoparticles are synthesized at different conditions of pH and bath temperature.

T (°C)	pH	Peaks Position CdS 2θ	C_s_	E_g_
50	6.8	25.06°, 26.85°, 28.48°, 44.15°, 48.31°, 51.89°	10	2.34
	9.4	25.26°, 26.92°, 44.34°, 52.23°	9	2.29
	10.1	25.26°, 44.10°, 52.04°, 54.86°	11	2.31
75	4.7	25.06°, 26.70°, 28.34°, 43.95°, 48.13°, 52.14°	10	2.39
	6.8	25.02°, 26.80°, 28.44°, 44.06°, 48.22°, 52.04°	10	2.36
	9.4	26.63°, 44.06°, 52.14°	7	2.27
	10.1	26.72°, 44.15°, 52.33°	9	2.34
90	4.7	25.06°, 26.62°, 28.28°, 36.58°, 44.06°, 48.10°, 52.26°	10	2.39
	6.8	25.30°, 26.63°, 28.47°, 44.10°, 52.04°	10	2.30
	9.4	25.30°, 26.63°, 28.29°, 44.06°, 52.24°	8	2.21
	10.1	26.72°, 44.15°, 52.33°	10	2.22

## Data Availability

The original data presented in the study are openly available on request from the corresponding author.

## Supplementary Materials

The following supporting information can be downloaded at: https://www.mdpi.com/article/10.3390/nano16010003/s1, Figure S1. CdS Clusters size analysis. Table S1. probability for CdS clusters between 20 to 80 nm.

## Abbreviations

The following abbreviations are used in this manuscript:

CdS	cadmium sulfide
SEM	scanning electron microscopy
XRD	X-ray diffraction
C_s_	crystallite size
FWHM	full width at half maximum
E_g_	band gap energy
