# Supplementary material for: From Structure to Optics: The pH-Temperature Interplay in Aqueous Solution CdS Nanoparticles
Source: Nanomaterials (Basel). 2025 Dec 19;16(1):3. doi: 10.3390/nano16010003 (PMC12788112; doi:10.3390/nano16010003)
Supplement: Supplementary file 1 [file nanomaterials-16-00003-s001.zip › nanomaterials-4017291-supplementary.pdf]

Supplementary Information

From Structure to Optics: The pH-Temperature Interplay in Aqueous Solution CdS Nanoparticles

Elvia Angelica Sanchez-Ramirez [1,*], Ramón Arellano-Piña [1], M. A. Hernandez-Perez [2], Simón Bello-Teodoro [3], Karol Karla Garcia-Aguirre [4], J. Sastré-Hernández [5] and J. R. Aguilar-Hernandez [5]

1 Departamento de Ingeniería Metalúrgica-UPIIZ, Instituto Politécnico Nacional, Zacatecas CP 98160, Mexico
2 Departamento de Ingeniería en Metalurgia y Materiales, ESIQIE, Instituto Politécnico Nacional, Ciudad de Mexico CP 07738, Mexico
3 Unidad Académica de Ingeniería I, Departamento de Metalurgia Extractiva, Universidad Autónoma de Zacatecas "Francisco García Salinas", Zacatecas CP 98160, Mexico
4 Departamento de Bioingenieria, Unidad Professional Interdisciplinaria de Ingeniería Campus Zacatecas, Instituto Politécnico Nacional, Zacatecas CP 98160, Mexico
5 Departamento de Física, Escuela Superior de Física y Matemáticas, Instituto Politécnico Nacional, Edificio 9, UPALM, Col. Lindavista, Ciudad de Mexico CP 07738, Mexico
* Correspondence: easanchez@ipn.mx

Figure S1 shows the probability density of the approximate diameters of the CdS clusters for all treatments. The data were obtained from the measurement of at least 250 CdS clusters by treatment to achieve a 90% confidence level. These measurements were performed using scanning electron microscopy (SEM) imaging at a magnification of 60,000X.

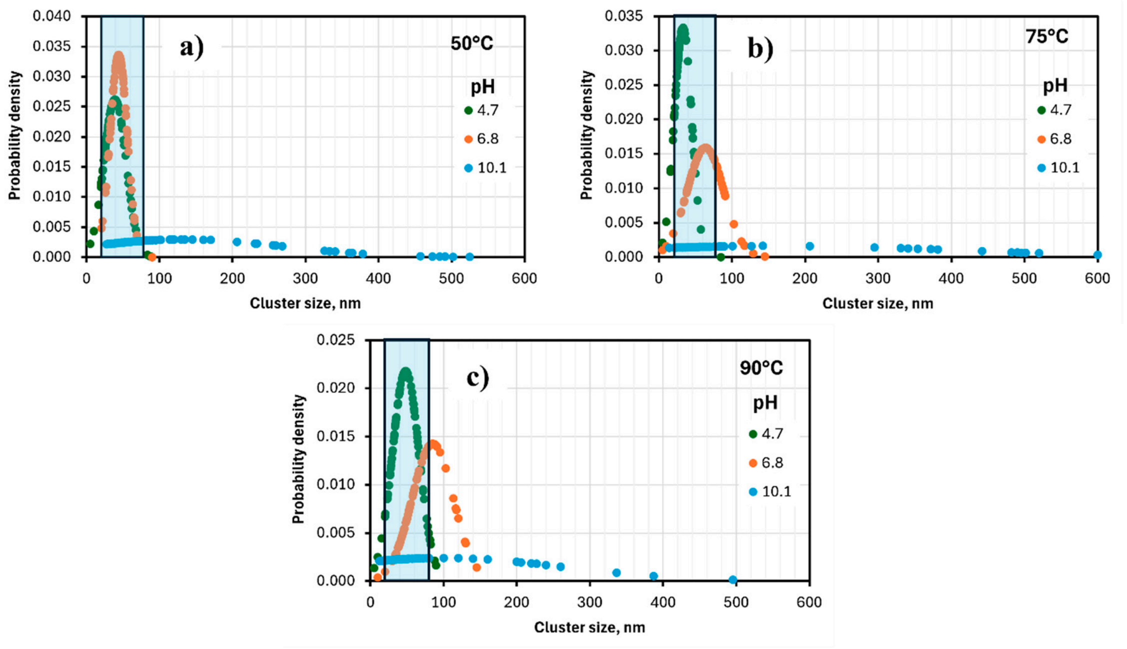

Figure S1. CdS Clusters size analysis

This figure shows that the variation in CdS cluster size is more sensitive to pH changes than to temperature changes during synthesis

Table S1. probability for CdS clusters between 20 to 80 nm

	Temperature			
pH	50°	75°	90°	Average
6.8	89%	86%	90%	88%
4.7	98%	70%	41%	70%
10.1	14%	9%	14%	12%

For example, at any temperature in the range of 50 °C to 90 °C and a pH between 4.7 and 6.8, at least 70% of the clusters will have sizes between 20 and 80 nm. In contrast, at alkaline pH (10.1), only 12% on average will be within the same size range. Therefore, the importance of pH for the correct formation of nanoparticle clusters can be deduced.
